# A comprehensive analysis of GAS2 family members identifies that GAS2L1 is a novel biomarker and promotes the proliferation of hepatocellular carcinoma

**DOI:** 10.1007/s12672-024-01083-0

**Published:** 2024-06-10

**Authors:** Ying-Ying Xu, Ru-Xue Bai, Qing-Rui Zhang, Shuang Zhang, Jun-Hai Zhang, Shi-Yu Du

**Affiliations:** 1 Department of Gastroenterology, China-Japan Friendship Hospital, No. 2, Yinghua East Street, Chaoyang District, Beijing, 100029 People’s Republic of China; 2 Graduate School, Beijing University of Chinese Medicine, Beijing, 100029 People’s Republic of China; 3 Graduate School, Chinese Academy of Medical Sciences & Peking Union Medical College, Beijing, 100730 People’s Republic of China

**Keywords:** GAS2 family, Hepatocellular carcinoma, Prognosis, Lipid metabolism, Tumor microenvironment, Immune cell infiltration

## Abstract

Hepatocellular carcinoma (HCC) is a common primary liver cancer with a high incidence and mortality. Members of the growth-arresting-specific 2 (GAS2) family are involved in various biological processes in human malignancies. To date, there is only a limited amount of information available about the expression profile and clinical importance of GAS2 family in HCC. In this study, we found that GAS2L1 and GAS2L3 were distinctly upregulated in HCC specimens compared to non-tumor specimens. Pan-cancer assays indicated that GAS2L1 and GAS2L3 were highly expressed in most cancers. The Pearson’s correlation revealed that the expressions of GAS2, GAS2L1 and GAS2L2 were negatively associated with methylation levels. Survival assays indicated that GAS2L1 and GAS2L3 were independent prognostic factors for HCC patients. Immune cell infiltration analysis revealed that GAS2, GAS2L1 and GAS2L3 were associated with several immune cells. Finally, we confirmed that GAS2L1 was highly expressed in HCC cells and its knockdown suppressed the proliferation of HCC cells. Taken together, our findings suggested the expression patterns and prognostic values of GAS2 members in HCC, providing insights for further study of the GAS2 family as sensitive diagnostic and prognostic markers for HCC.

## Introduction

Hepatocellular carcinoma (HCC) is a highly prevalent malignant disease worldwide, with the sixth highest diagnostic rate and the third highest mortality rate among cancers [1]. In modern clinical practice, the key risk factors for HCC are increasingly associated with a sustained virological response to hepatitis C and suppressed hepatitis B virus on therapy [2, 3]. The clinical signs of HCC do not appear in the early stages of the disease; rather, they become apparent in the late stages, which leads to unsatisfactory results in terms of curative treatment [4–6]. The diagnosis and treatment of HCC have come a long way in recent years; yet, the prognosis for the vast majority of patients remains dismal, with only around 10% of patients surviving for 5 years or more [7, 8]. Additionally, a high rate of disease recurrence is a contributing factor in the majority of HCC patients having poor outcomes. Despite the fact that medications like sorafenib and immune checkpoint inhibitors have the potential to enhance patients' prognoses, the outcome is still not good enough [9, 10]. Because of this, more sensitive biomarkers are urgently required for the diagnosis and prognosis of individuals with HCC.

Members of the family known as growth-arrest-specific 2 (GAS2) are structurally similar to spectraplakins because they have actin-binding regions in addition to MT-binding regions, but they do not have plakin domains [11]. There are four different members that make up the GAS2 family: GAS2, GAS2-like 1, GAS2-like 2, and GAS2-like 3. (GAS2L1, GAS2L2 and GAS2L3, respectively) [12]. The gene GAS2L2 is expressed in a wide variety of human organs. In Xenopus embryos, it was discovered that it inhibits cell division and has a role in the process of apoptosis (cell death) [13]. G2L1 is engaged in the process of limiting the formation of red blood cells and is expressed in the testis as well as the brain [14]. This process occurs downstream of the signaling of the thyroid receptor. Skeletal muscle is the only tissue in which GAS2L2 is expressed, however very little is known about the function of this gene [15]. GAS2L3 can be found in a wide variety of cell types, and our research has shown that it has the ability to bind to actin as well as MTs [16]. Additionally, it is selectively increased during mitosis, and it makes a contribution to the regulation of the cell cycle. Aneuploidy was observed after knocking down GAS2L3 expression in human BJ fibroblasts and HCT116 cells. This finding suggests that dysregulation of GAS2L3 may be involved in the development of tumors. It has been suggested that these members influence the cytoskeleton system in such a way as to be responsible for cellular polarization, motility, or centrosome dynamics. In the past several years, growing researches have shown the deregulation of GAS2 family in a number of different cancers [17–19]. However, the function of the GAS2 family in HCC has been rarely reported.

There is a growing body of research suggesting that the tumor microenvironment (TME) plays an important part in the genesis of tumors [20, 21]. The interactions between cancer cells and their supporting cells defined the malignant properties of the tumor, such as immortalization, immune escape, and resistance to apoptosis [22, 23]. Cancer cells collaborated with their supporting cells to interact with one another. As a result, TME has the potential to affect both the therapeutic effects and the long-term survivals for cancer patients [24]. The TME is mostly composed of structural cell elements such as resident stromal cells and immune cells that have been recruited [25]. In the meantime, research indicated that stromal cells may play a role in distant metastasis, but the mechanisms underlying this role are not entirely understood. It is important to highlight that a significant number of researchers have focused their attention on the roles that immune cells in TME play in the developments of tumors [26, 27]. According to the findings of several studies, the tumor-infiltrating immune cell (TIC) in the TME may be a helpful measure of the treatment’s effectiveness. In addition to this, an enhanced tumor-infiltrating CD8 +lymphocyte demonstrated to be a sensitive biomarker for HCC patients after undergoing neoadjuvant chemotherapy.

In the first part of this investigation, we investigated the expression pattern and prognostic significance of the members of the GAS2 family in HCC. The next step is for us to investigate further the link between members of the GAS2 family and the microenvironment of tumors. The opportunities presented by our results to further our understanding of the genesis of HCC and to create new therapeutic agents are being taken advantage of.

## Materials and methods

### Data collection

The TCGA-LIHC and the cBioportal for Cancer Genomics were able to provide the clinical data and level 3 mRNA expression data from the 374 LIHC samples and the 50 normal control samples respectively. Since the data were obtained by downloading them from a database that was open to the public, further ethical approval was not required for this study.

### Comparison of the expression of GAS2 family in HCC specimens and normal tissues

First, we used the Wilcoxon test to select potentially differentially expressed genes(DEGs) between 374 HCC cases and 50 normal control samples to obtain GAS2 family members with prognostic value. Adjusted P values were calculated using the BH16 approach after the Wilcoxon tests and the fold change calculation. Criteria for |log2FC|> The value of 1.0 was chosen, along with an adjusted P value of f 0.05. Using Perl, we were able to determine the expressions of the GAS2 family by analyzing the expressions of the entire genome. The expression of the GAS2 gene family was then compared in HCC using the “corrplot” tool in R.

### Correlation between expression and methylation of the GAS2 family in HCC

To obtain information on the methylation levels of cg sites in the gene promoter regions of differentially expressed GAS2 members in HCC tissues, we used the GDC Data Transfer Tool suggested by TCGA to retrieve data from Illumina HumanMethylation 450 K. The association between methylation and GAS2 expression in HCC was then further investigated using the corrplot program.

### Analysis of GAS2 expression in cancers

Differential GAS2 expression between tumors and their matched normal counterparts was gleaned from TCGA and the Genotype Tissue Expression (GTEx) studies. In total, 53 human normal tissues from almost 1000 individuals have been researched through the NIH Common Fund’s GTEx tissue repository and data resource. When it came to picking parameters, we settled on plotting log2 (TPM+ 1) transformed expression data.

### COX regression analysis

For univariate COX regression, the R programming language with the survival package installed was utilized. The plot displayed the top 16 genes in univariate COX, arranged according to their p values, which ranged from modest to high.

### Tumor-infiltrating immune cells (TICs) profile

The computational method known as CIBERSORT was used to estimate the TIC abundance profile in each and every tumor sample. This was followed by quality filtering, which resulted in the selection of only 421 tumor samples with a p value of < 0.05 for the subsequent analysis.

### Cell culture and treatment

The normal liver cell line (L02) and HCC cell lines (SMMC7721, Huh7, HepG2, HCCLM3, and SK-HEP-1) were obtained from the Biological Research Institute of the Chinese Academy of Sciences. The use of short tandem repeat profiling allowed for the verification of each cell line. The cells were grown in high-glucose DMEM medium (Invitrogen) supplemented with 10% fetal bovine serum (Invitrogen) in a culture chamber containing 5% carbon dioxide at a temperature of 37 degrees Celsius. The siRNAs (si-GAS2L1 and si-NC) were purchased from JiMa Biological Corporation (Suzhou, Jiangsu, China). Lipofectamine 2000 reagent kits were used to transfect cells according to the kits' instructions.

### Quantitative real-time polymerase chain reaction (qRT-PCR)

The total RNA from cells was extracted with 1 ml TRizol reagent (Invitrogen, USA). After that, a NanoDrop was utilized in order to ascertain the concentration as well as the purity of the RNA, and a cDNA preparation was carried out using a random primer reverse transcription kit (Thermo, USA). 2 µl cDNA was used in a 20 µl qPCR reaction. the SYBR GREEN kit (TaKaRa, Japan) was used to analyze GAS2L1 expression levels. The cycling condition was: 95 ℃ for 10 min and 40 cycles of 95 ℃ for 10 s, and 58 ℃ for 30 s. GAPDH was selected as internal reference for GAS2L1. The expression of the desired gene was calculated using the qRT-PCR experiment data by counting the number of cycles per cycle (2Ct technique). RT-PCR for GAS2L1 was carried out with the forward primer 5ʹ- GACACGCTGGAGCATTACCTG-3ʹ and reverse primer 5ʹ- TGTGGAGAAAAGGTGCAGACC-3ʹ. GAPDH forward: 5ʹ- GGAGCGAGATCCCTCCAAAAT-3ʹ and reverse: 5ʹ- GGCTGTTGTCATACTTCTCATGG-3ʹ.

### CCK-8 assay

The CCK-8 assay was used, following the technique provided by the manufacturer, to determine the level of cell proliferation. After transfection, HCCLM3 and SK-HEP-1 cells were grown in 96-well plates for the incubation step. Each sample was given an additional 90 µl of fresh culture media and 10 µl of CCK-8 solution after 12, 24, 48, and 72 h had passed. After that, the transfected HCCLM3 and SK-HEP-1 cells were allowed to rest at 37 degrees Celsius for two hours. A microplate reader set to 450 nm was utilized to calculate the optical density value.

### Statistical analysis

R software, version 4.0.3, was used to perform the analysis on all data. The Student’s paired two-tailed t-test was applied to analyze the statistical differences between the groups. In this study, we compared categorical variables using either the chi-squared test (χ2 test) or the Fisher exact test. A Kaplan–Meier analysis was carried out to compare the overall survival curves of the various patient groups; a log-rank test was then performed to evaluate the statistical significance of the findings. If the p-value is less than 0.05, it indicates that the differences are statistically significant.

## Results

### *Expression *pattern* of GAS2 *m*embers in HCC*

**Fig. 1:**
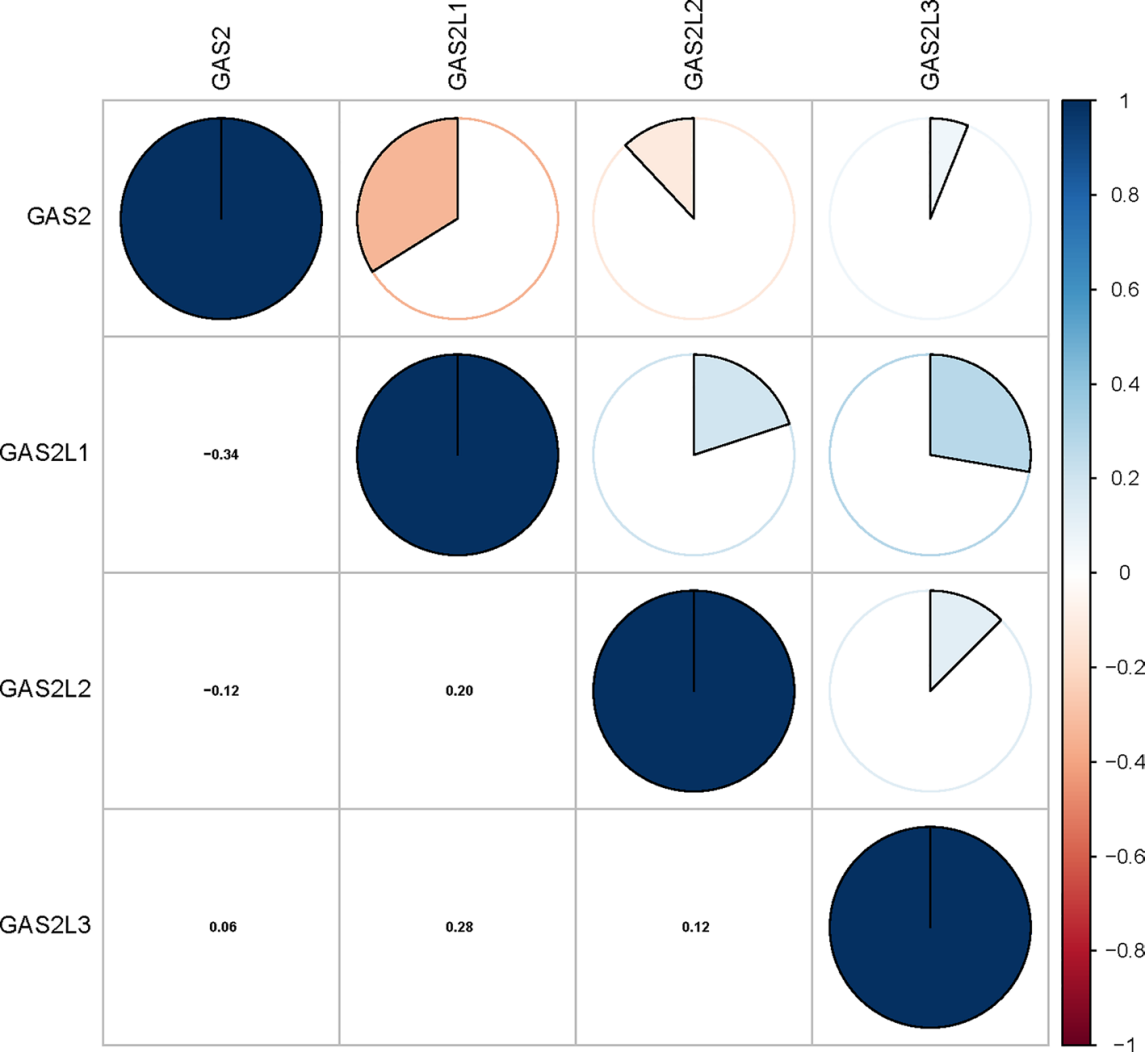
Associations between GAS2 family members

To begin, the expressing data of mRNAs for GAS2 members (GAS2, GAS2L1, GAS2L2, and GAS2L3) from 374 HCC cases and 50 non-tumor samples that came from TCGA were collected using Perl software. The Pearson’s correlations of GAS2 family genes were examined. We observed that the GAS2 family genes exhibited a significant with each other (Fig. 1).

**Fig. 2:**
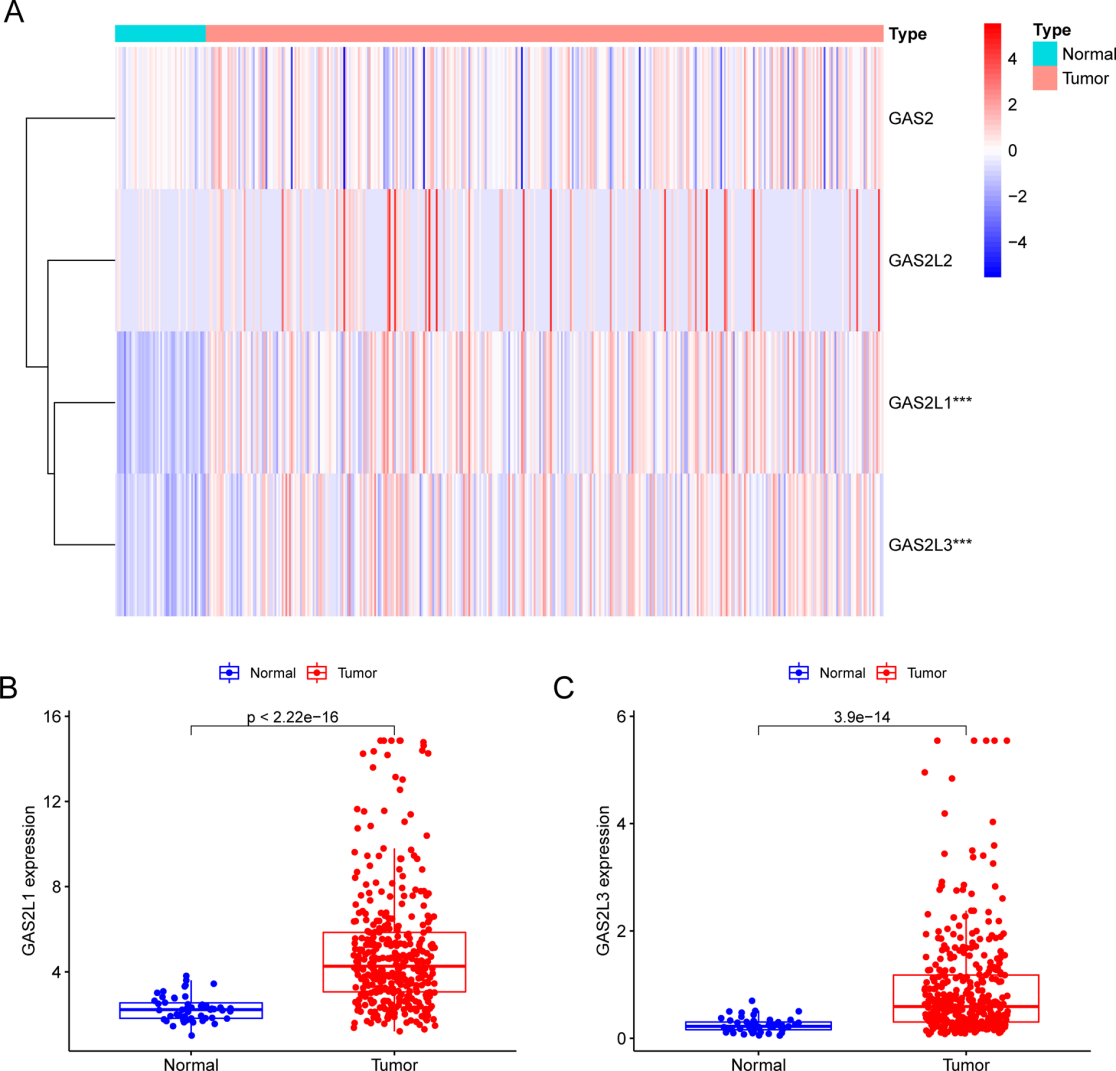
The expression pattern of GAS2 family members in HCC specimens and non-tumor specimens based on TCGA datasets were shown in (**A**) Heat Map. (**B** and **C**)The expression of GAS2L1 and GAS2L3 were distinctly increased in HCC samples

After that, the limma package was used to conduct an analysis on the differentially expressed GAS2 members, and the results were then visualized. making use of the pheatmap package, as demonstrated in Fig. 2A. In addition, GAS2L1 and GAS2L3 were distinctly upregulated in HCC tissues compared to non-tumor specimens (Fig. 2B and C).

### *Pan*-*cancer* assays of GAS2L1 and GAS2L3

**Fig. 3:**
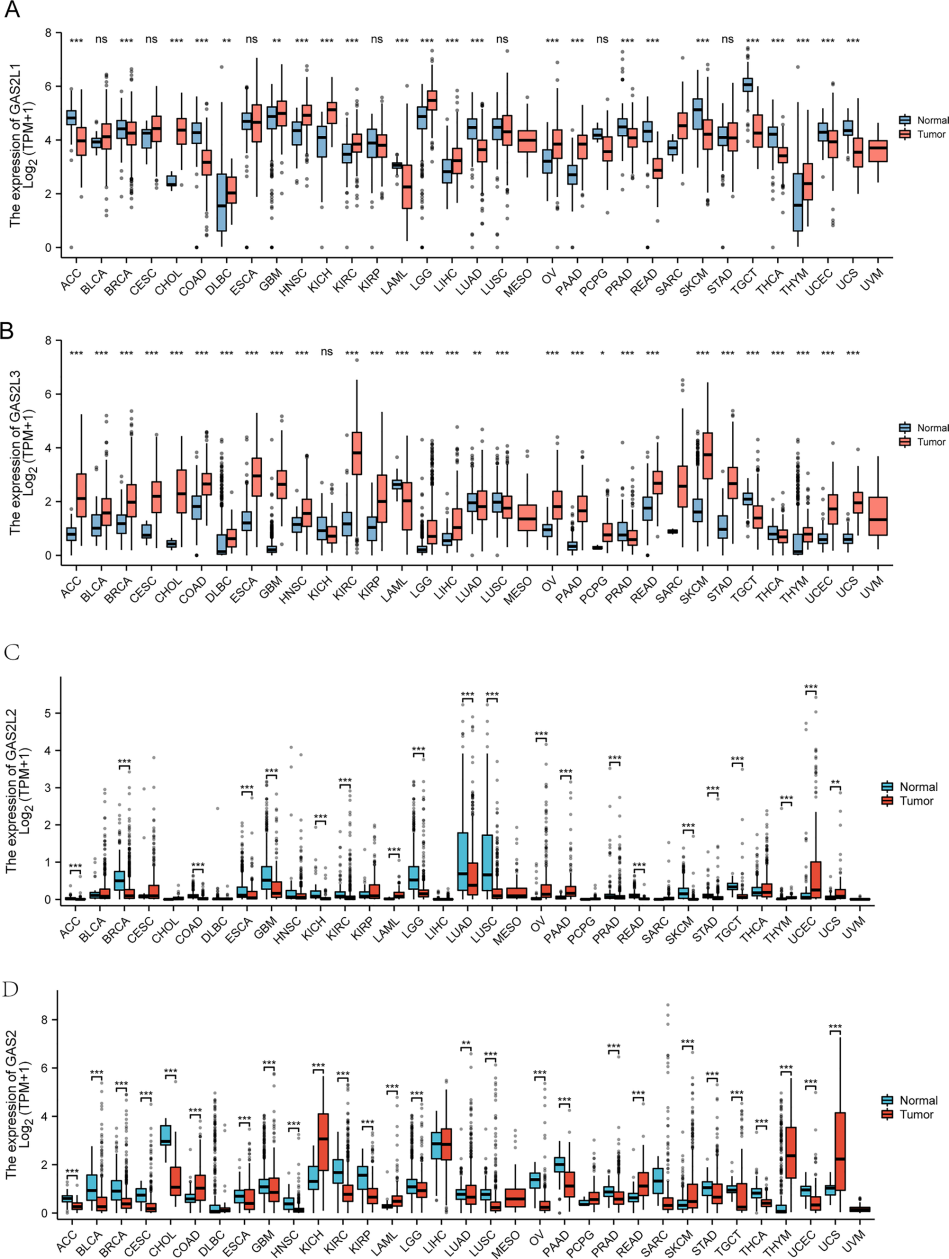
Pan-cancer analysis of (**A**) GAS2L1, **B** GAS2L3, **C** GAS2L2 and **D** GAS2

To delve into the potential function of GAS2L1 and GAS2L3 in tumors, we performed pan-cancer assays and found that the expression of GAS2L1 was distinctly increased in CHOL, DLBC, GBM, HNSC, KICH, KIRC, LGG, LIHC, OV, PAAD and THYM, while decreased in ACC, BRCA, COAD, LAML, LUAD, PRAD, READ, SKCM, TGCT, THCA, UCEC and UCS (Fig. 3A). Moreover, we observed that the expression of GAS2L3 was distinctly increased in ACC, BLCA, BRCA, CESC, CHOL, COAD, DLBC, ESCA, KIRP, KIRC HNSC, GBM, LGG, LIHC, OV, PAAD, PCPG, READ, SKCM, STAD, THYM, UCEC and UCS, while was distinctly decreased in LAML, LUAD, LUSC, PRAD, TGCT and THCA (Fig. 3B). In addition, we also can observed that GAS2L2 and GAS2 exhibited a dysregulated level in many types of tumors (Fig. 3C and D).

### Correlation of GAS2 expression and methylation in HCC

**Fig. 4:**
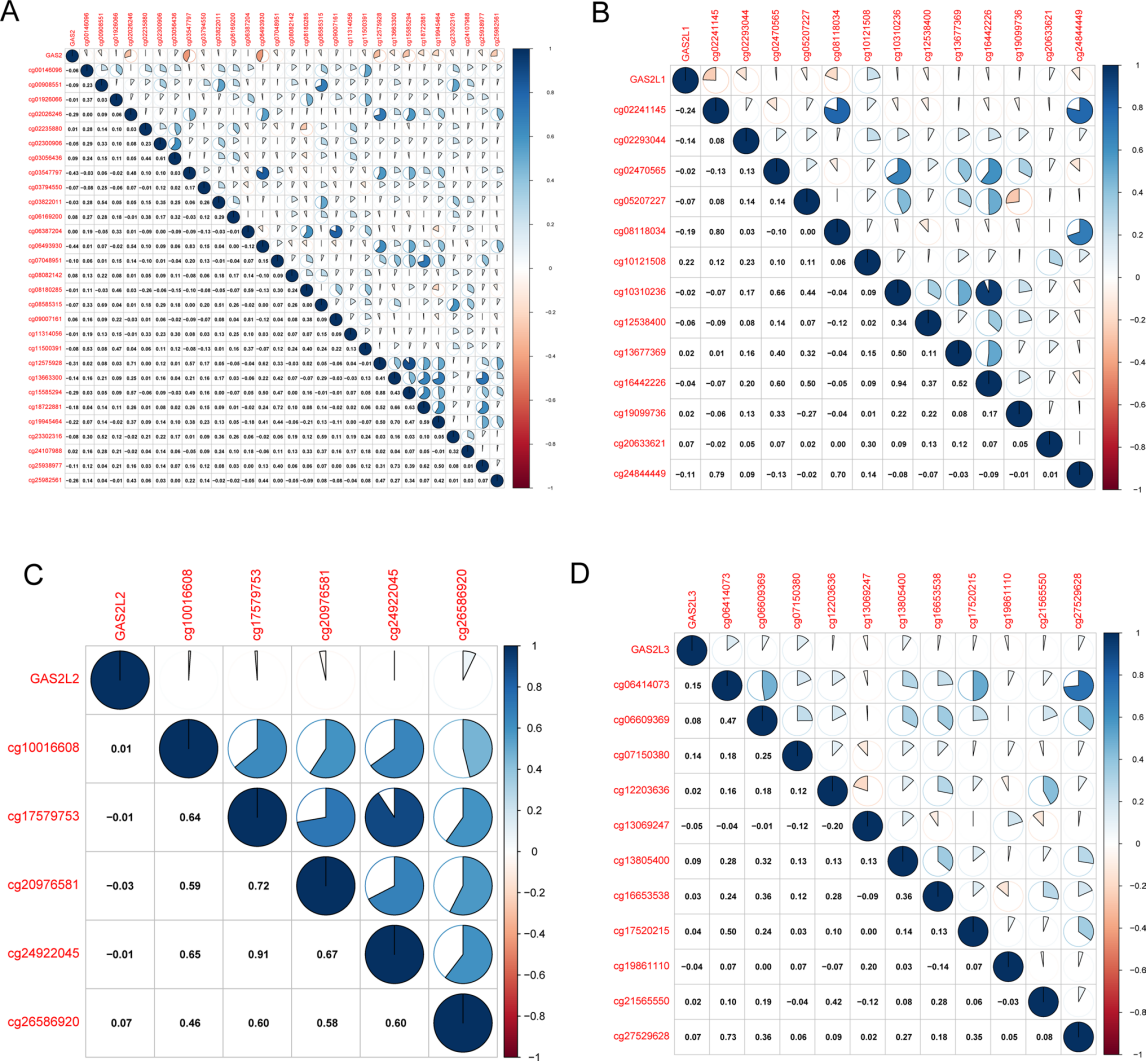
Pearson’s correlation between methylation levels and expressions of GAS2 family members, including (**A**) GAS2, **B** GAS2L1, **C** GAS2L2 and **D** GAS2L3

During the course of human cancer, methylation of gene promoter regions is one of the most common processes that impacts the expressions of various genes. The findings of Pearson's correlation demonstrated that a negative association exists between the levels of methylation and the expressions of GAS2, GAS2L1, and GAS2L2 (Fig. 4A–D). Based on our data, a negative relationship was observed between the expressions of GAS2 genes in HCC and their methylation station.

### The prognostic values of GAS2 Members in HCC patients

**Fig. 5:**
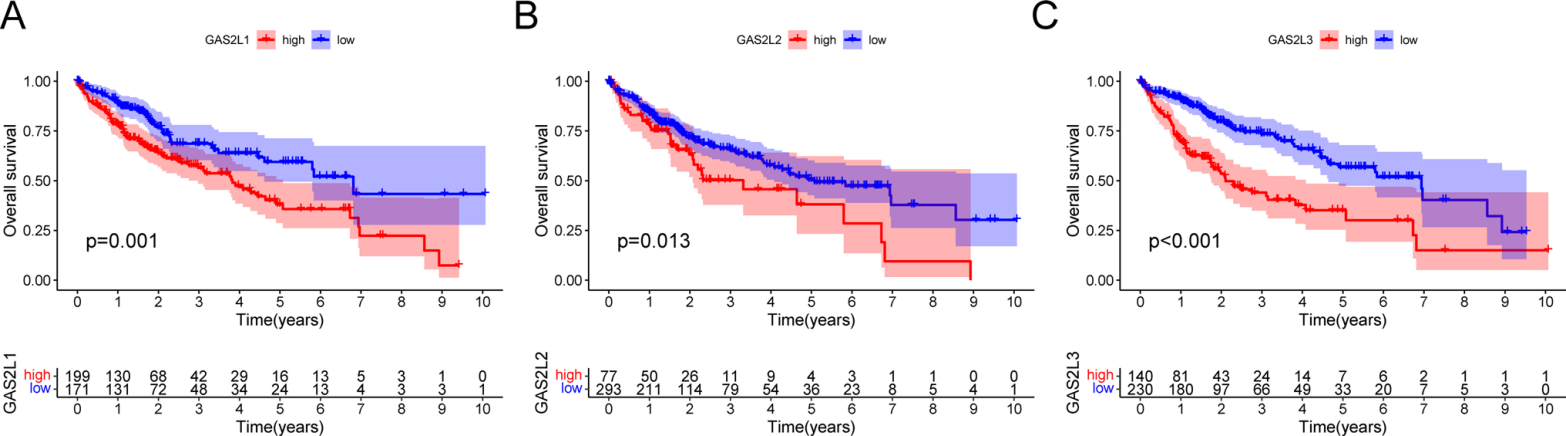
Kaplan–Meier curves of the overall survival of 374 HCC patients based on the expression of (**A**) GAS2L1, **B** GAS2L2 and **C** GAS2L3

**Fig. 6:**
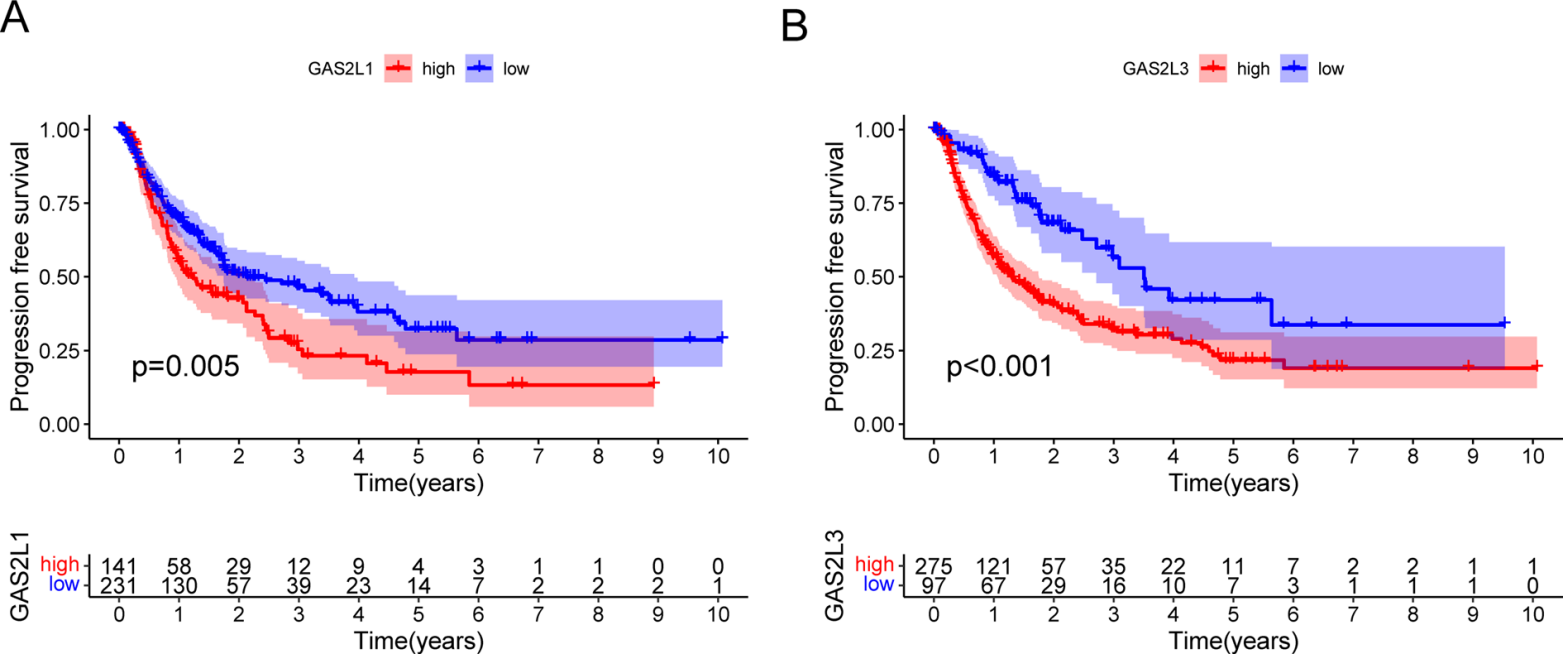
Kaplan–Meier curves of the progression free survival of 374 HCC patients based on the expression of (**A**) GAS2L1 and (**B**) GAS2L3

**Fig. 7:**
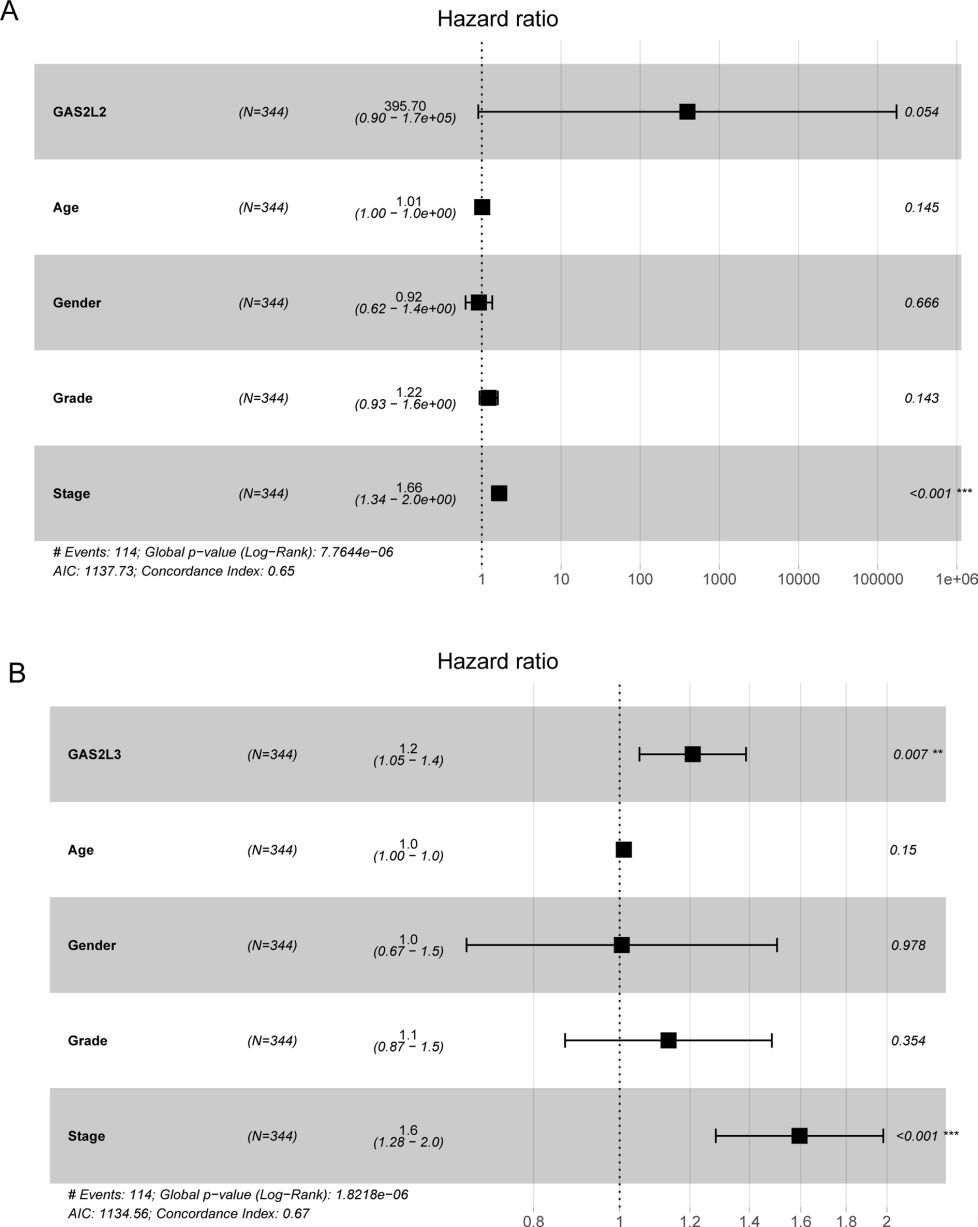
Multivariate analyses of (**A**) GAS2L2 and (**B**) GAS2L3 in HCC patients

Then, we performed Kaplan–Meier analysis to analyze the prognostic values of GASL2 Members in HCC patients. The results showed that high expression of GAS2L1, GAS2L2 and GAS2L3 predicted a shorter overall survival of HCC patients (Fig. 5A-C). Moreover, we found that patients with high expressions of GAS2L1 and GAS2L3 were related to a shorter progression-free survival (Fig. 6A and B). To further investigate whether GAS2L1 and GAS2L3 were independent prognostic factors for HCC. In a multivariate analysis, we found that high GAS2L1 (Fig. 7A) and GAS2L3 (Fig. 7B) expressions was independent prognostic factors for HCC patients.

### Correlations between TIICs and GAS2 members

**Fig. 8:**
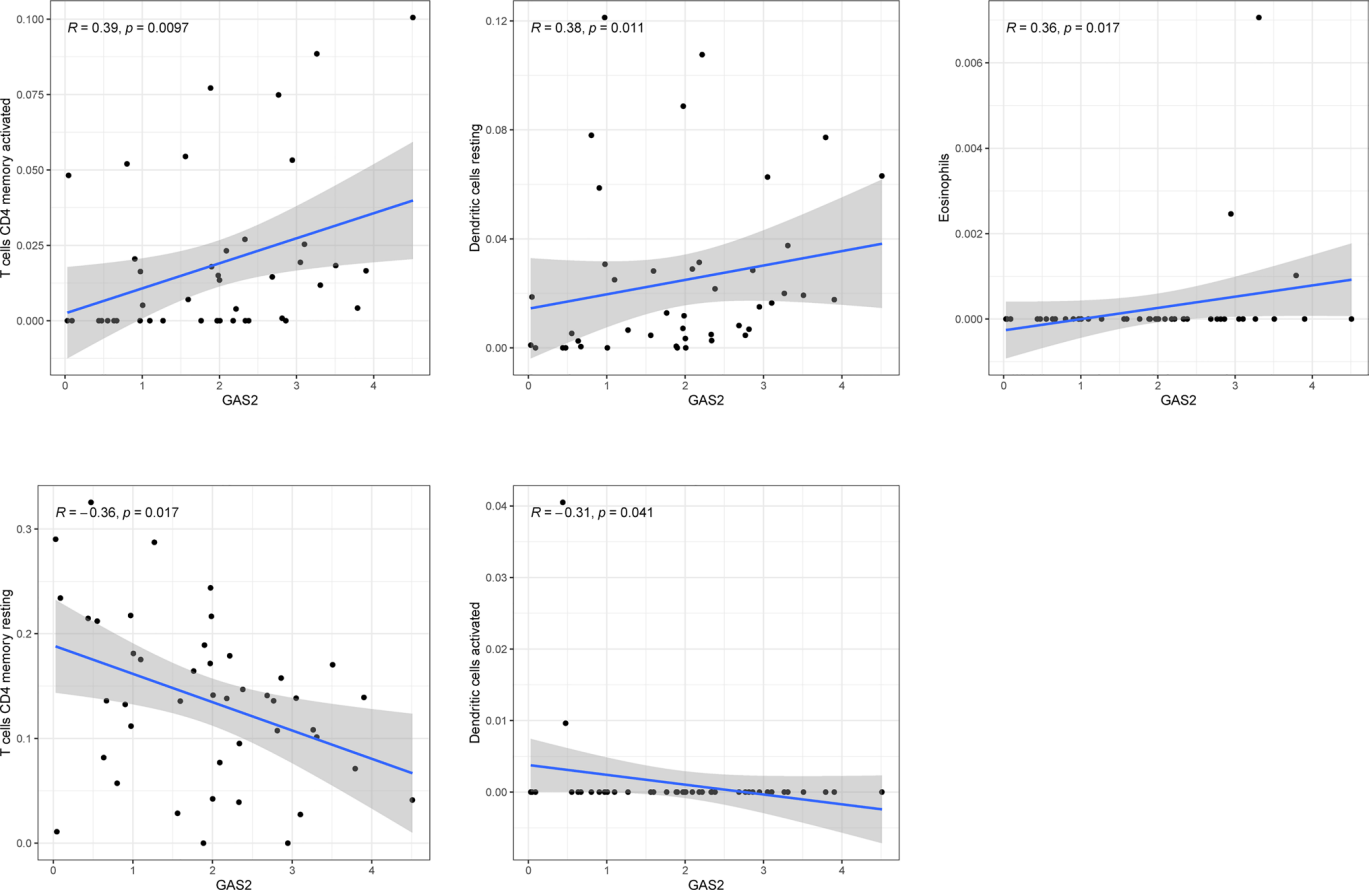
The correlations between GAS2 and immune infiltration levels in HCC

**Fig. 9:**
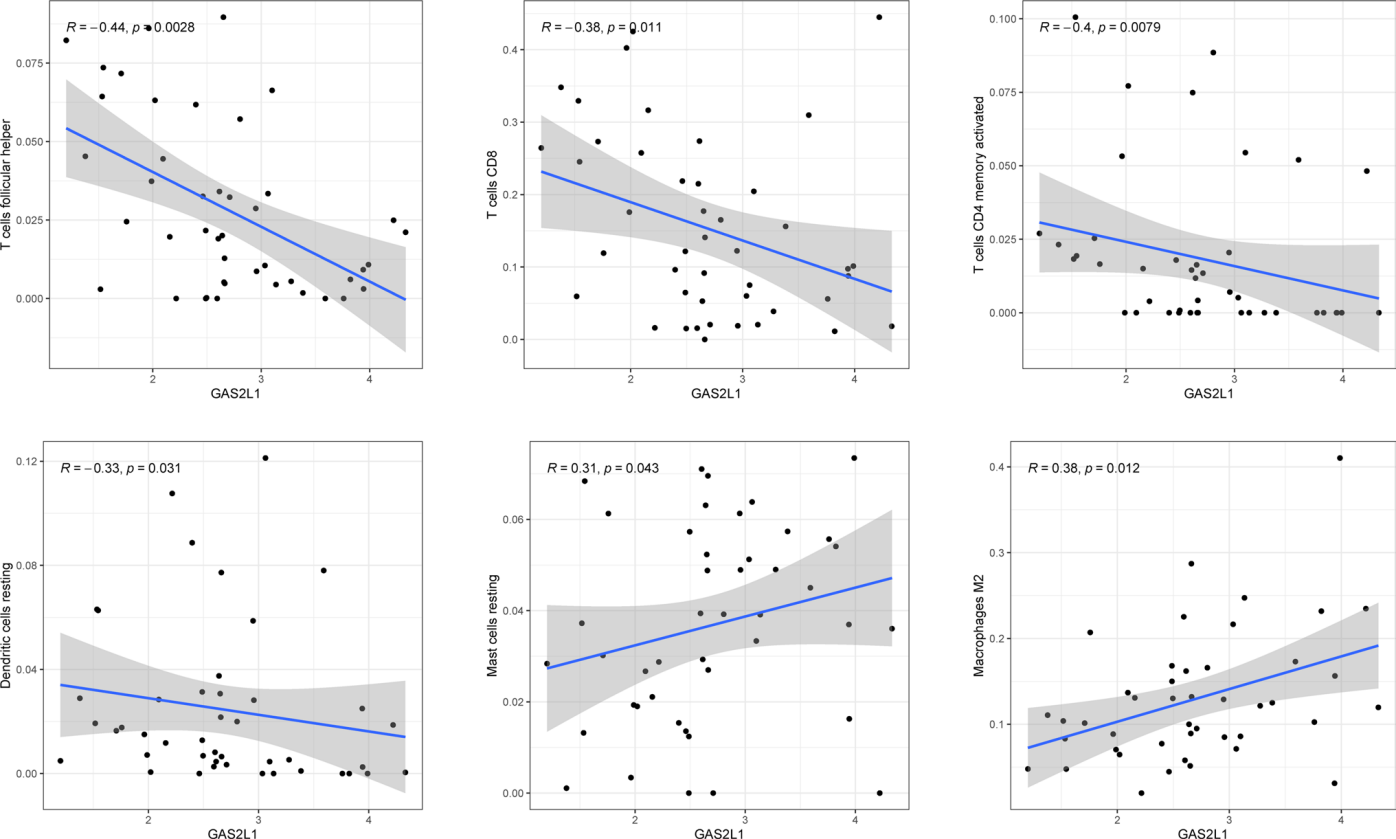
The correlations between GAS2L1 and immune infiltration levels in HCC

**Fig. 10:**
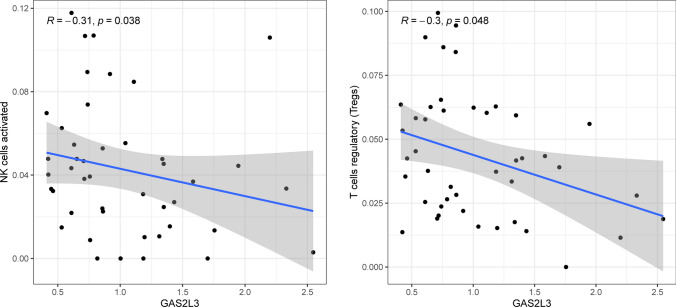
The correlations between GAS2L3 and immune infiltration levels in HCC

The CIBERSORT algorithm was used to analyze the fraction of tumor-infiltrating immune subsets to gain further confirmation of the association between GAS2 members and the immunological microenvironment. The results of correlation assays revealed that the expression of GAS2 was positively related to activated CD4 memory T cells, resting dendritic cells, and eosinophils, while negatively associated with activated dendritic cells and resting CD4 memory T cells (Fig. 8). The expression of GAS2L1 was negatively related to resting dendritic cells, activated CD4 memory T cells, CD8 T cells, and follicular helper T cells, while positively related to M2 macrophages and resting mast cells (Fig. 9). In addition, the expression of GAS2L3 was negatively related to activated NK cells and regulatory T cells (Fig. 10).

### GAS2L1 may regulate the progression of Lipid metabolism

**Fig. 11:**
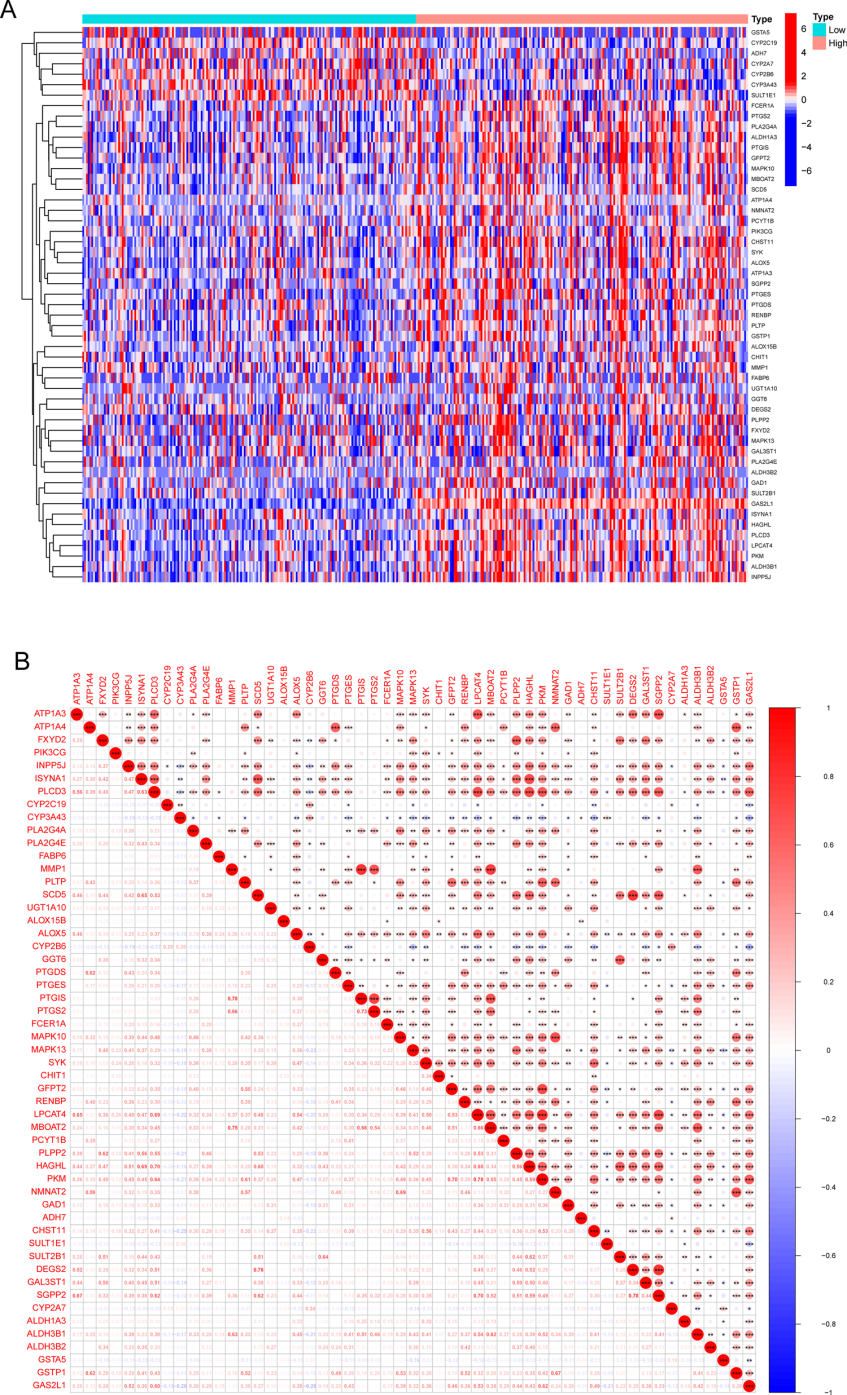
The association between GAS2L3 expression and lipid metabolism related genes. **A** 53 lipid metabolism related DEGs between high-GAS2L1 expression group and low-GAS2L1 expression group. **B** The correlation analysis between GAS2L1 expression and 53 DEGs

**Fig. 12:**
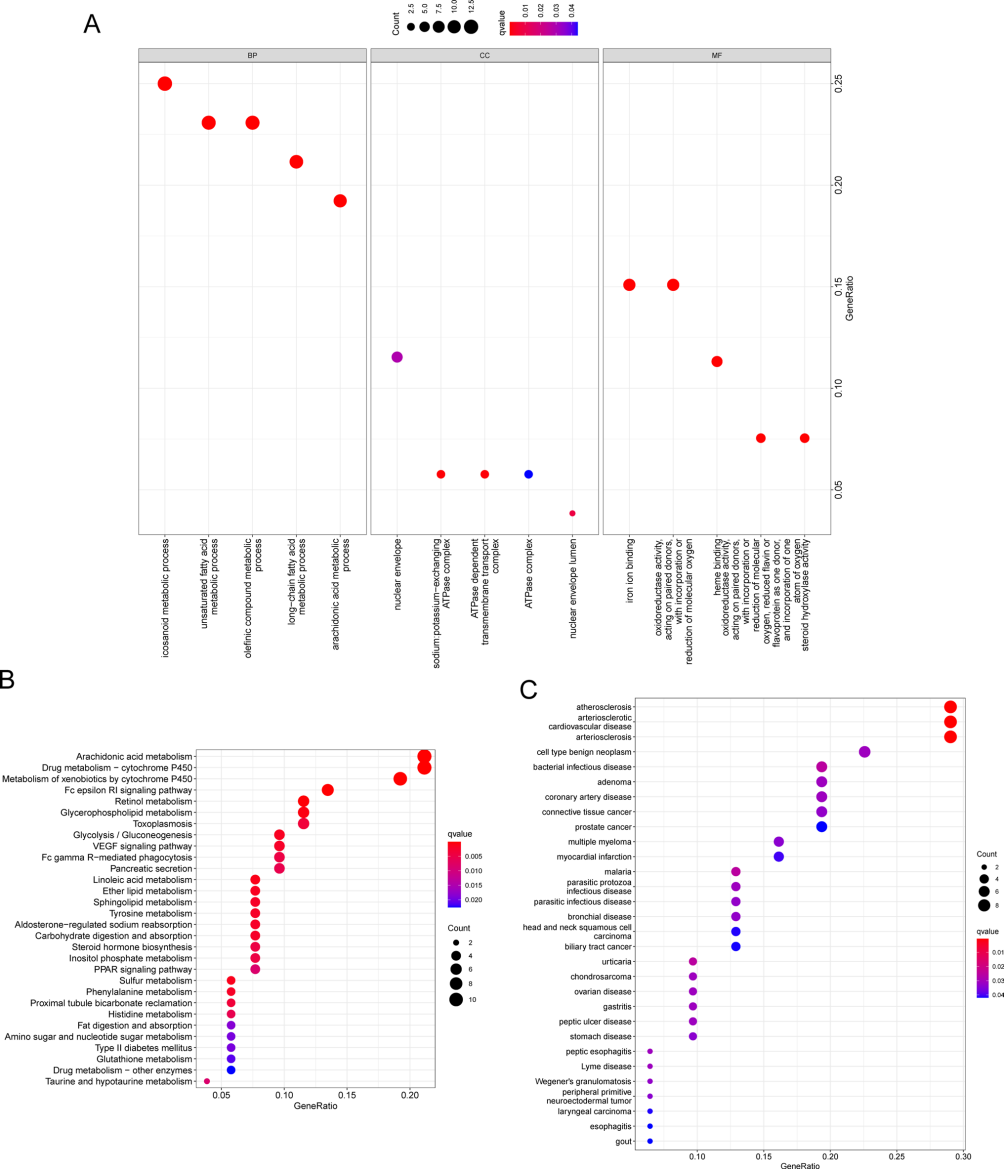
Enrichment analysis of 53 DEGs. **A** GO function assays. **B** KEGG pathway assays and **C** Disease ontology enrichment analysis

To explore the potential function of GAS2L1 in HCC progression, we divided all HCC samples into two groups(High and Low) based on the mean expression of GAS2L1. Then, we found that 53 differently expressed genes (DEGs) between high- GAS2L1 expression group and low-GAS2L1 expression group (Fig. 11A). The correlation between GAS2L1 and all DEGs were shown in Fig. 11B. Then, we performed functional enrichment analysis using the 53 DEGs. The results of GO analysis suggested that 53 DEGs were mainly associated with icosanoid metabolic process, unsaturated fatty acid metabolic process, olefinic compound metabolic process, long-chain fatty acid metabolic process, nuclear envelope, sodium:potassium − exchanging ATPase complex and iron ion binding (Fig. 12A). KEGG assays revealed that the 53 DEGs were mainly associated with Arachidonic acid metabolism, Drug metabolism-cytochrome P450, Metabolism of xenobiotics by cytochrome P450, Fc epsilon RI signaling pathway, Retinol metabolism (Fig. 12B). DO assays revealed that the 53 DEGs were mainly associated with atherosclerosis, arteriosclerotic cardiovascular disease, arteriosclerosis and cell type benign neoplasm (Fig. 12C). Our findings suggested GAS2L1 may play an important role in the progression of Lipid metabolism.

### The potential function of GAS2L1 in HCC progression

**Fig. 13:**
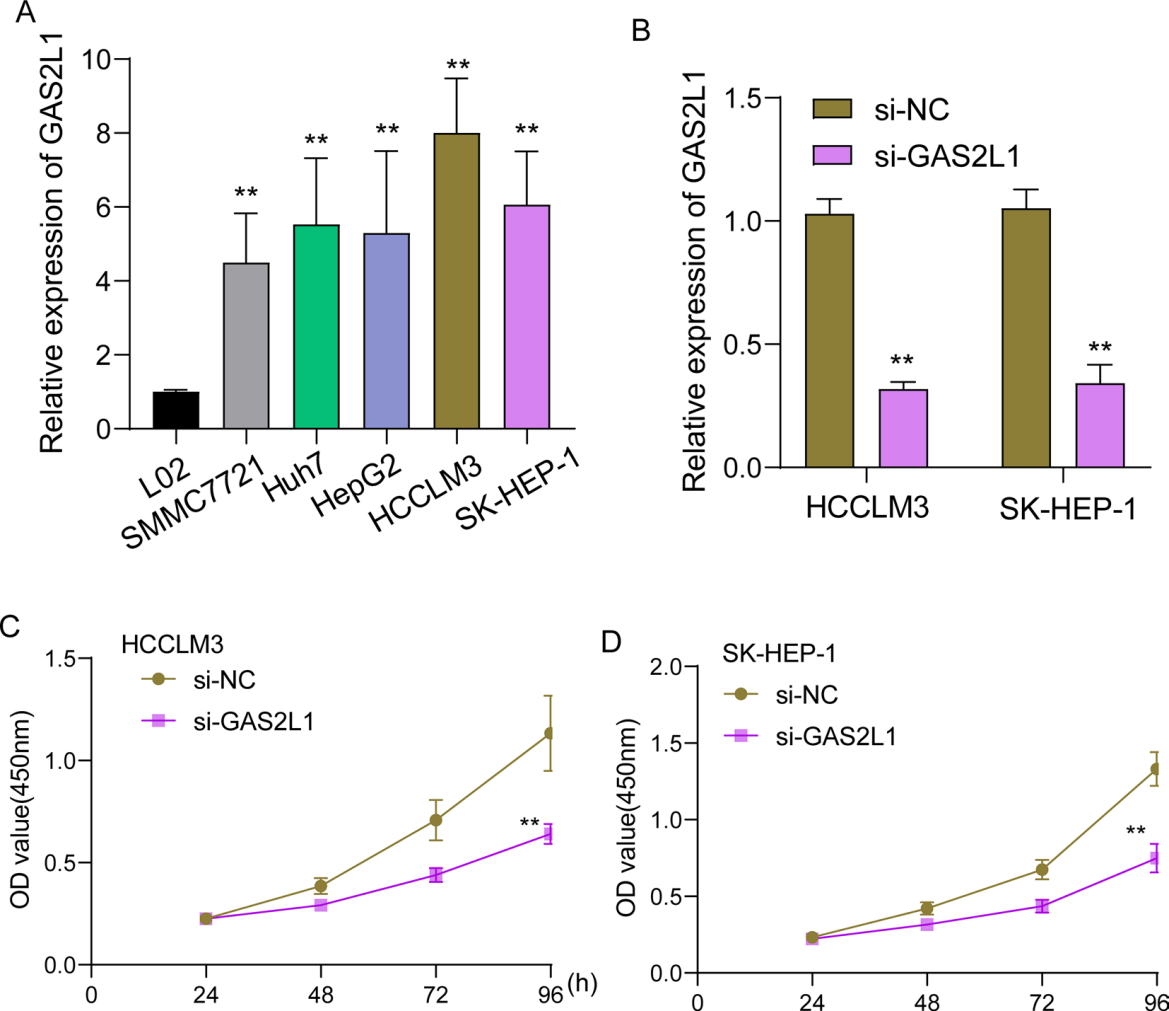
Knockdown of GAS2L1 suppressed the proliferation of HCCLM3 and SK-HEP-1 cells. **A** RT-PCR for the expression of GAS2L1 in five HCC cells and LO2 cells. **B** Detection of knockdown efficiency of GAS2L1 by RT-PCR. **C** and **D** CCK-8 assays were applied to examine the function of GAS2L1 knockdown on the proliferation of HCCLM3 and SK-HEP-1 cells. **p < 0.01

Then, we performed RT-PCR to examine the expression of GAS2L1 in HCC cells. As shown in Fig. 13A, we found that GAS2L1 expression was distinctly increased in five HCC cells compared with LO2 cells. Moreover, the transfection of si-GAS2L1 confirmed the knockdown of GAS2L1 expression in HCCLM3 and SK-HEP-1 cells (Fig. 13B). The results of CCK-8 analysis indicated that knockdown of GAS2L1 distinctly inhibited the proliferation of HCCLM3 and SK-HEP-1 cells (Fig. 13C and D).

## Discussion

HCC is the most prevalent primary malignancy of the liver, accounting for about 90% of all malignant cases [28]. It is also the most curable form of primary liver cancer. The fact that the development of HCC is a multistep process that is also a multigene alteration-induced malignancy with a high level of heterogeneity has been well reported [29–31]. Additionally, the fact that HCC has a high degree of heterogeneity has also been extensively documented. It has been determined that hepatitis B, hepatitis C, alcoholism, steatohepatitis, and obesity are all etiologic factors that contribute to the disease [32, 33]. Recent studies at the molecular level have indicated that specific gene mutations play an important part in the progression of HCC.

In recent years, several studies have indicated that GAS2 Family members played an important role in the progression of several tumors. For instance, Zhu et al. reported that in compared with mononuclear cells found in the peripheral blood, CLIC4 and GAS2L1 were found to have higher levels of expression in pancreatic CTCs (PBMCs). Validation of the overexpression of GAS2L1 was accomplished through additional analysis of PBMCs. An increase in the identification rate of CTCs in PDAC can be achieved by a combinatorial approach that makes use of both GAS2L1 and EPCAM expression in GEMM as well as in patient samples. As a result, GAS2L1 is being considered for use as a potential new biomarker of pancreatic cancer CTCs [34]. The expression of the GAS2 protein was shown to be decreased in HCC tissues compared to normal tissues [19]. Compared to normal brain tissues, the GAS2L3 gene was shown to have significantly higher levels of expression in glioma tissues, as demonstrated by Zhao et al. Patients diagnosed with glioma had a worse prognosis if they had a high expression of the GAS2L3 gene. It is possible that GAS2L3 has a role in the development of glioma through having an effect on a variety of biological processes. These processes include cell division, the attachment of the cytoskeleton to cells, and cell adhesion. In addition, the results of our cellular experiments revealed that a GAS2L3 gene that is highly expressed contributes to the increased proliferation and migration of glioma cells [35]. In addition. Zhu and his group reported that overexpression of GAS2 had an inhibitory effect on the proliferation of HCC cells expressing wide-type p53, whereas knockdown of GAS2 had the opposite effect and encouraged the proliferation of hepatocytes. In addition, GAS2 overexpression inhibited the transition from the G1 phase to the S phase of the cell cycle and arrested more G1 cells, particularly the elevation of sub-G1 cells. The induction of apoptosis by GAS2 was dependent on p53, and this dependency was exacerbated by the addition of etoposide. In this study, we firstly performed Integrative analysis to explore the expression pattern of GAS2 family members, and found that the expression of GAS2L1 and GAS2L3 was distinctly increased in HCC specimens compared with non-tumor specimens. In addition, pan-cancer assays confirmed that GAS2L1 and GAS2L3 exhibited a higher level in many types of tumors. Our findings suggested GAS2L1 and GAS2L3 may serve tumor promotors in HCC. Then, we analyzed the prognostic value of GAS2 family members and confirmed that high GAS2L1 and GAS2L3 expressions were independent prognostic factors for HCC patients. Importantly, we performed loss-of-function experiments and confirmed that knockdown of GAS2L1 distinctly suppressed the proliferation of HCCLM3 and SK-HEP-1 cells. Our findings suggested GAS2L1 and GAS2L3 may be novel prognostic biomarkers for HCC patients.

The identification of GAS2L1 as a biomarker for HCC holds significant potential for enhancing patient diagnosis, prognosis, and treatment strategies. Integrating GAS2L1 expression levels into diagnostic protocols could improve the early detection of HCC, complementing current methods such as imaging techniques and alpha-fetoprotein (AFP) levels, which often lack sensitivity and specificity. Additionally, GAS2L1 could serve as a prognostic marker, offering insights into disease progression and patient outcomes. Stratifying patients based on GAS2L1 expression levels could help predict tumor aggressiveness and recurrence likelihood, guiding monitoring and follow-up strategies. Furthermore, GAS2L1 could inform treatment strategies by highlighting specific molecular pathways involved in HCC, potentially leading to targeted therapies. Personalized treatment plans could be developed based on GAS2L1 expression, with high levels indicating the need for more aggressive interventions. Future research should validate GAS2L1 in larger cohorts and explore its biological role in HCC, with clinical trials assessing the efficacy of GAS2L1-targeted therapies. Overall, incorporating GAS2L1 into clinical practice could enhance early detection, predict patient outcomes more accurately, and develop targeted therapies, ultimately improving the management and survival of HCC patients.

There is accumulating evidence to suggest that aberrant DNA methylation plays a critical part in the initiation and development of HCC [36, 37]. In the course of our investigation, we began by determining, with the help of Pearson coefficients, whether or not the methylation status of GAS2 family members may have an effect on the expression of GAS2 family members. In HCC tissues, there was a significant inverse relationship between the degree to which GAS2, GAS2L1, and GAS2L2 were methylated and the amount of mRNA that was expressed for GAS2, GAS2L1, and GAS2L2. This inverse association could very well explain why GAS2, GAS2L1, and GAS2L2 are expressed at such low levels in HCC tissues.

Growing body of evidence pointed to the role of the TME in tumor formation, particularly in HCC. HCC is an inflammatory tumor, and its immunosuppressive microenvironment can develop immunological tolerance in a variety of ways [38]. This is due to the fact that HCC is an immunosuppressive tumor. Immunotherapy that stimulates an immune response that is directed specifically against tumors offers fresh hope for the treatment of HCC [39]. HCC's microenvironment is made up of a variety of cellular and non-cellular elements, including tumor infiltrating lymphocytes, tumor-associated fibroblasts, myeloid-derived suppressor cells (MDSCs), tumor-associated neutrophils, tumor-associated macrophages and other cellular components. Studies have shown that TICs in the TME play a significant role in HCC progression and act as a prognostic indicator [40, 41]. In this study, we found that the expression of GAS2 was positively associated with T cells CD4 memory activated, Dendritic cells resting and Eosinophils, while negatively associated with Dendritic cells activated and T cells CD4 memory resting. The expression of GAS2L1 was negatively associated with Dendritic cells resting, T cells CD4 memory activated, T cells CD8, T cells follicular helper, while positively associated with Macrophages M2 and Mast cells resting. In addition, the expression of GAS2L3 was negatively associated with NK cells activated and T cells regulatory. Our findings suggested GAS2 family members may play an important role in TME.

Lipid metabolism refers to the processes of synthesis, breakdown, and utilization of fats in the body [42]. Fats are an important form of energy storage, and their synthesis and breakdown in the body are regulated by various biochemical reactions and mechanisms [43]. There is a certain relationship between lipid metabolism and HCC. HCC is a malignant tumor that occurs in liver tissue. Disruptions in lipid metabolism may have an impact on the development and progression of HCC [44]. The liver is one of the main organs involved in lipid metabolism. Prolonged high-fat diet and unhealthy lifestyle can lead to the accumulation of fat in the liver, resulting in fatty liver [45]. Fatty liver is a risk factor for the development of HCC, as it can progress to liver fibrosis, cirrhosis, and ultimately HCC. The accumulation of fat in the liver can lead to the activation of chronic inflammation and immune responses. These inflammatory and immune responses may promote the development of liver cancer through various mechanisms, including promoting tumor cell proliferation, inhibiting tumor cell apoptosis, and promoting angiogenesis. Some metabolites produced during the process of lipid metabolism may play a role in the development of HCC. For example, certain fatty acid metabolites such as leukotrienes, lipid peroxidation products, and inflammatory mediators may participate in the occurrence and progression of HCC by activating cellular signaling pathways, damaging DNA, and inducing genetic mutations [46, 47]. In this study, we performed functional enrichment analysis and found that GAS2L1 may an important role in the progression of fatty acid metabolic process. In addition, we provided evidences that GAS2L1 may influence the activity of arachidonic acid metabolism, retinol metabolism and linoleic acid metabolism. Our findings suggested GAS2L1 may act as a tumor promotor in HCC progression via regulating the progress of Lipid Metabolism.

Nevertheless, there are some general limitations in our study. First, for the purpose of validating the predictive importance of GAS2 family members, a larger patient sample size of HCC is necessary. Second, we were unable to examine the expression profile of GAS2 family members in serum and plasma samples from patients diagnosed with HCC. It is an effective way to examine responses to treatments in real time by detecting markers in the serum and plasma samples of HCC patients. Third, we were unable to further investigate the connection between GAS2 family members and tumor immunocytes because the database prevented us from doing so. We can only investigate the potential mechanism in our future work.

## Conclusion

Overall, the findings from our bioinformatics analysis suggested that GAS2L1 and GAS2L3 had distinct expression patterns in HCC. It has been discovered that GAS2L12 and GAS2L3 are independent prognostic variables for patients with HCC. According to the results of our research, GAS2L12 and GAS2L3 are both useful prognostic biomarkers for individuals suffering from HCC as well as viable targets for cancer immunotherapy.

## Data Availability

The original data are provided by the corresponding author upon request without any hesitation.
